# Camp Typhoid Fever

**Published:** 1864-02

**Authors:** Wm. S. Edgar

**Affiliations:** Surgeon 32d Regt. Ill. Vols.


### CHICAGO MEDICAL EXAMINER.

N. S. DAVIS, M.D., Editor.

VOL. V. FEBRUARY, 1864. NO. 2.

### Original Contributions.

### ARTICLE VI.

### CAMP TYPHOID FEVER.

By WM. S. EDGAR, Surgeon 32d Regt. Ill. Vols.

Having enjoyed some opportunities for witnessing the ravages of this form of continued fever, as it exists in the central armies of the United States, I propose briefly to record some of my observations and conclusions concerning the primary causes and pathology; also, the complications and after pathology as determined by *post mortem* examinations.

Typhoid, like other fevers, is ushered in with a chill, but often so slight as to have escaped the particular notice of the patient, unless his attention be called to it, when he may say he has been chilly repeatedly the past day or two. I observe nothing peculiar in these chills or rigors diagnostic of typhoid.

These cases occurring in camp, are first noticed as being dull of perception, lying about, averse to exercise, hardly know what to complain of, but indicate debility. When closely questioned, they often say they "are sick all over;" appetite impaired, headache, confusion of mind, apparently sleeping much, but not refreshed, disturbed dreaming, or wakeful, with apprehension of some impending calamity.

During the exacerbation of fever, a burning heat of the skin is noticed; when bathed with perspiration the heat is not reduced, as is usual with other fevers.

Thus far tne patient is reported ailing, unfit for duty, perhaps for three days, when he is admitted to hospital, and the attending surgeon, with the condition above described before him, proceeds to investigate the history of the case, to complete or establish the diagnosis if possible. The patient may be a stalwart lumberman from the forests of Michigan, Wisconsin, or Minnesota, whose brain never reeled nor muscles relaxed before with fever. He has but recently exchanged the pleasures and hilarity of the social board and family hearthstone, the associations of his childhood and youth, for the company of men, and mostly, if not entirely, strangers.

The rifle is placed in his hands, and the stern reality of exposure to wounds and death is upon him for the first time, (for the greatest prevalence of this fever is soon after the mustering in of raw troops,) I say the good cheer of the home table has suddenly been exchanged for the rough fare and associates of the camp. The joys and hilarity of the evening village party, or family fireside, is exchanged for the solitary tramp of the picket guard, far from home and friends; and we notice that with the less intelligent—those who read and write least—these moral agencies are most powerful.

I will call attention in this connection to the unprecedented mortality in some of the recently recruited black regiments, reaching, in some instances, one hundred a month, the majority of this disease occurring in their native climate, and while better fed and clothed (indeed their clothing has been too heavy) than their previous custom; their accustomed hilarity they miss. The duties and perils of their new life have engrossed their attention and depressed their spirits. Separated from their families, sweet-hearts, and homes, the merry song and exciting dance, the banjo and bones, that rang through the spacious cotton gin or sugar mill, no more impart that healthful and vitalizing activity to every function, particularly the brain, so indispensable to perfect digestion.

Instead, the deadly rifle and sabre are placed in their hands, constantly reminding them of the personal danger of their new employment, thereby depressing their spirits, and imparting a grave, thoughtful deportment previously unknown to them.

Or our patient may have rank, and more responsible duties, and the nerve power may have become exhausted by long-continued, anxious labor, night and day, until his strength gives way, and he is compelled to seek recuperation through a long and weary process of repair. His sleep is but little, which does not refresh, as it is broken and disturbed with fever. I have a case in mind of a surgeon who had the charge of many wounded at the battle of Shiloh, and passed many days without sleep or rest, until he was relieved by the distribution of his patients in general hospitals, when he immediately sank down with a slow typhoid fever, and inability to sleep, from which he did not recover for many months.

It matters little from what cause the nerve power fails, whether by exhausting labor and watching, or depressed by protracted grief, or fear, whether by moral or physical causes, if the nerve power fails, the functions one after another fail.

True, we may meet with cases of typhoid fever without these concomitants, and with altogether different history; still, that some subtle agencies have primarily disturbed the cerebral function, we think will be manifest on careful inquiry.

Having sufficiently noted, as we trust, the evidence of the primary disturbance of the nerve power in this fever, I propose to consider some of the immediate consequences and complications, among which, to be first noticed, is the function of digestion, not so much in the stomach as in the duodenum, as the food taken into the stomach is broken down, rendered liquid by the gastric juice, and passes to the next organ, where the secretions failing, or being unhealthy, the farther elaboration of nutrient elements fails, and the chylopoietic vessels are not supplied. Instead thereof, chemical agencies supervene, forming acrid irritating excrementitious matters, which, exciting increased peristaltic actions, are hurried forward through the small intestines to tarry longer in the pouches of the colon, exciting congestion, and finally inflammation and ulceration of bowel, if the evil be not timely arrested.

The waste of the circulation being imperfectly replenished by the food taken, together with the effete and irritating matters retained in the circulation from the general failure of the glands to eliminate the same, abundantly explains the excitement of the heart and arteries, and the creation of the continued fever, as also its persistent character.

From this period we may have gradual decay; one function after another fails. Those of the skin, kidneys, and liver claim our early and special attention. As the case progresses to the condition just described, it is common to find rose-colored pimples, first on the forehead and breast, gradually extending over the body, (but this symptom is not constant, as some have erroneously claimed,) more or less incoherent talking, delirium, and the irritation already referred to in the bowels. The last is indicated by the dry, brown, cracked tongue, with red edges, offensive, watery ejections from the bowels, tenderness on firm pressure in various parts of the abdomen, which often becomes tympanitic.

The fever, if influenced by malarial complication, will observe some variation, exhibiting on alternate days exacerbations and remissions.

Typhoid pneumonia, commonly called winter fever, in the West and South, is not included in this description.

To recapitulate, the symptoms on which our diagnosis of camp typhoid fever is formed, are first the partial failure of the cerebral function, and as a consequence, the subsequent failure, in some degree, of all the functions of the body, the particular symptoms of which have already been mentioned.

To meet the first indication we would use early and persistently a nerve stimulant. Arnica, sulph. ether, (or Hoffman's anodyne, which is preferable,) from four to six stimulating doses every twenty-four hours. To promote digestion we would restrict the diet to the simplest, most nutritious, and easily assimilated articles, in limited quantities, beef tea, arrow-root, tapioca, and the like.

To aid the skin and kidneys to resume their functions, use warm baths, with the occasional internal use of diaphoretics and diuretics; to promote the secretions of the liver and glands generally, a dose or two of calomel, mercury and chalk, or blue-mass, after the warm bath, or washing the body in warm water by sections (if the bath cannot be had); but the indiscriminate use of mercurials to cure this fever is unwise and even pernicious. If we have reason to apprehend complication from malaria (as is often the case) we give from three to four grains of quinine every two or three hours, until fifteen or twenty grains have been administered, during the remission, if any is observable, repeating the quinine in five or six days if the fever continues.

To obviate serious disease of the mucous membrane of the bowels, we resort early to the use of mild laxatives, repeated during the progress of the disease, and to the same end, and to blunt the acrimony of the secretions allow free use of mucilaginous drinks, which also comforts the patient by allaying his thirst. If the bowel affection becoms a serious complication, as too often occurs, notwithstanding the most judicious management of the case, and inflammatlon or ulceration is threatened; to relieve the tympanotic distension, we would cleanse the lower bowels first, with warm water injections, and after placing our patient on his right side, introduce a well oiled elastic bougie to the descending colon, whereby large quantities of flatus may be evacuated, greatly to the relief of the patient. It is usual to fail in this simple operation from neglect to place the patient on his right side, as it is obvious that if the patient is on his left side when the instrument is passed, the gas will occupy the highest point of the colon, viz: the ascending colon which may not be reached by the instrument, hence the failure. But if the patient be on his right side, the gas will ascend to the descending portion of the colon which brings it within reach.

This farther suggests the importance of the patient's position when local applications are made to the mucous membrane of the lower bowels, and which I apprehend receives but little attention from many practitioners. For instance, if a clyster is designed to evacuate the bowels, after it is administered, the patient should always be directed to lie on his left side which position throws forward the contents of the ascending and transverse colon, thus favoring the evacuation, but if it is desirable that the material thrown into the bowel should remain as long as possible, if the patient be placed on his right side the hydrostatic pressure of the transverse colon is removed, and retention favored. An anodyne administered in this way at night, often assists materially in procuring rest. Astringents, vegetable or mineral, to check the diarrhœa are usually pernicious.

The patient's mind should be prepared from the outset for a protracted illness, yet the most hopeful prognosis should be persistently adhered to from the beginning to the end, both by medical advisers and attendants, which is of the utmost importance. No doubt or question of the final recovery should be intimated.

The ulceration of the mucous membrane of the bowels, and disease of Peyers' glands, so common where the case runs to a fatal termination, we regard as purely incidental, originating in the long continued presence of acrid irritating secretions and combinations in the bowels, rather than idiopathic.

I have endeavored in this brief sketch to show the importance of introducing into our camps, as prophylactics, *exhilarating mental influences*, more hilarity, fun, and amusements of whatever kind may be found practicable, such as games, music, dancing, horse-racing, &c.

It has already been stated that those who are not sufficiently intelligent to read and write, suffer most from this disease. The fact itself sufficiently intimates that such as are able to profit by it should be provided with cheerful and entertaining reading, and as far as possible the discipline of the camp should cultivate the intelligence of the soldier and cheerful employment of the mind.

If I have contributed a single suggestion of value, either for the prevention or in the treatment of this most prevalent and destructive disease, I will be pardoned by my professional brethren for this intrusion.

